# Safety and probiotic characterization of *Lacticaseibacillus rhamnosus* SAL2: insights from integrated genomics and functional validation

**DOI:** 10.3389/fmicb.2026.1767191

**Published:** 2026-02-27

**Authors:** Jie Sun, Zhixue Li, Xinyi Lin, Jinlian Huang, Yamin Chen, Yuzhen Xu, Yanping Xu, Xueying Cheng, Ben Liu, Yongzhi Lun

**Affiliations:** 1 Key Laboratory of Screening and Control of Infectious Diseases, Quanzhou Medical College, Fujian Provincial University, Quanzhou, China; 2 Department of Microbiology, School of Basic Medical Sciences, Guilin Medical University, Guilin, China

**Keywords:** antioxidant activity, *Lacticaseibacillus rhamnosus*, probiotic properties, safety assessment, whole genome sequencing

## Abstract

**Background:**

Integrated genomic-phenotypic frameworks provide a more comprehensive approach to probiotic characterization, resolving longstanding ambiguities in strain safety assessment. Crucially, extracellular metabolites–not cell-bound components–emerge as dominant mediators of bacterial antioxidant activity, thereby refining the mechanistic understanding of microbial reactive oxygen species mitigation.

**Methods:**

This study evaluated the probiotic potential and safety of *Lacticaseibacillus rhamnosus* SAL2 using whole-genome sequencing and functional validation. Hybrid Illumina/PacBio sequencing enabled complete *de novo* assembly, annotation, and pathogenicity assessment of this independently isolated strain. We benchmarked *L. rhamnosus* SAL2 against the reference strain *L. rhamnosus* GG through multidimensional assessment of safety and probiotic characterization. Antioxidant capacity was further evaluated through H_2_O_2_ tolerance assays and free radical scavenging tests.

**Results:**

The genome of *L. rhamnosus* SAL2 comprises a single circular chromosome of 2,989,570 bp with no plasmids. Annotation using the KEGG, GO, COG, CAZy, and TCDB databases revealed that the majority of genes are involved in carbohydrate metabolism, cellular nitrogen compound metabolic process, carbohydrate transport and metabolism, glycoside hydrolases, and primary active transporters, respectively. Virulence factors were primarily limited to immune evasion mechanisms. *L. rhamnosus* SAL2 lacked hemolytic activity and was susceptible to multiple antibiotic classes. The strain exhibited high viability under simulated human gastrointestinal conditions while displaying strong mucosal adhesion potential. Notably, both intact cells and cell-free fermentation supernatants of *L. rhamnosus* SAL2 exhibited significant antioxidant activity. When comprehensively benchmarked against the classic probiotic reference strain *L. rhamnosus* GG, *L. rhamnosus* SAL2 matched its core safety profile and probiotic properties while demonstrating quantitatively superior antioxidant activity.

**Conclusion:**

*Lacticaseibacillus rhamnosus* SAL2 showed safety and probiotic characteristics comparable to the reference strain, with enhanced antioxidant performance. These findings highlight its potential as a probiotic candidate for developing microecological preparations or functional foods, particularly due to its exceptional gastrointestinal tolerance, adhesive capacity, and free radical scavenging efficacy.

## 1 Introduction

The growing consumer demand for natural health-promoting agents has significantly propelled the probiotic industry. *Lacticaseibacillus rhamnosus*, a facultatively anaerobic, acid-tolerant, non-spore-forming Gram-positive bacterium, is widely recognized for its antioxidant properties, immunomodulatory effects, and ability to balance gut microbiota (Huang et al., 2023; Lee et al., 2024; Xu et al., 2024). Isolated from fermented foods, humans, and animal intestines, this species is listed as a safe food-grade microorganism by China’s National Health Commission. Concurrently, the paradigm for probiotic strain evaluation is shifting from a primary focus on phenotypic characteristics to a deeper understanding of genomic foundations and mechanistic actions. This evolution underscores the critical need for rigorous, multi-layered safety assessments that move beyond traditional methods. The advent of high-throughput whole-genome sequencing has become indispensable for modern safety evaluations, enabling *in silico* detection of virulence determinants and metabolic pathways that underpin both safety and functionality. However, probiotic properties are highly strain-specific, necessitating individual assessment even within the same species.

Despite the established framework for probiotic evaluation, a significant gap remains in the integrated application of high-resolution genomics and targeted functional assays for the characterization of newly isolated strains. Furthermore, while the antioxidant activity of various Lactobacilli has been documented, the primary mechanisms-whether by cell-bound components (e.g., surface-layer proteins) or secreted extracellular metabolites-remain inconsistently characterized across studies, leading resulting in ambiguous structure-function interpretations (Ge et al., 2021; Zhou et al., 2022; Qin et al., 2024; Lee et al., 2025).

This study aimed to comprehensively evaluate the probiotic potential and safety of the independently isolated strain *Lacticaseibacillus rhamnosus* SAL2. We employed a hybrid sequencing strategy for complete genome assembly and annotation, followed by multidimensional *in vitro* characterization. Our objectives were to: genomically assess its safety profile; functionally validate key probiotic attributes, including gastrointestinal tolerance, mucosal adhesion, and antibiotic susceptibility; quantitatively evaluate its antioxidant capacity and elucidate the primary contributor (cells vs. metabolites) to this activity; and benchmark all characteristics directly against the reference strain *L. rhamnosus* GG. This integrated genomics-phenomics approach was designed to robustly validate *L. rhamnosus* SAL2 as a promising probiotic candidate with potential applications in functional foods and microbiota-targeted formulations.

## 2 Materials and methods

### 2.1 Strains and reagents

The experimental strain *Lacticaseibacillus rhamnosus* SAL2 (CGMCC No. 32935) was isolated, identified, and deposited in the China General Microbiological Culture Collection Center (Lin et al., 2018). The reference strain *Lacticaseibacillus rhamnosus* GG was procured from Hangzhou Bio-SCI Biotechnology Co., Ltd., (Hangzhou, China).

De Man, Rogosa and Sharpe (MRS) broth medium, MRS agar medium, blood agar plates, and bovine bile salt were supplied by Qingdao Hope Biotechnology Co., Ltd., (Qingdao, China). Sodium chloride (NaCl), concentrated hydrochloric acid (HCl), and hydrogen peroxide (H_2_O_2_) were acquired from Sinopharm Chemical Reagent Co., Ltd., (Shanghai, China). Trypsin, pepsin, xylene, 1,1-diphenyl-2-picrylhydrazyl (DPPH), o-phenanthroline, ferrous sulfate (FeSO_4_), Tris buffer, and pyrogallol were sourced from Beijing Solarbio Science & Technology Co., Ltd., (Beijing, China) and Shanghai Macklin Biochemical Co., Ltd., (Shanghai, China).

### 2.2 Strain activation

Cryopreserved *L. rhamnosus* strains stored at −80°C were aseptically inoculated into MRS broth medium and incubated at 37°C with agitation (150 rpm) for 18 h. The revived cultures were streaked onto MRS agar plates using a sterile inoculating loop and incubated anaerobically at 37°C for 48 h. Following three successive subcultures under anaerobic conditions, a single representative colony exhibiting typical morphology was selected and transferred to fresh MRS broth for incubation at 37°C for 18 h to prepare the seed culture.

### 2.3 Genomic library construction

*Lacticaseibacillus rhamnosus* SAL2 starter cultures were centrifuged (8,000 × *g*, 10 min, 4°C), and the cell pellets were washed twice with sterile saline (0.85% NaCl). The concentrated biomass (1 mL) was flash-frozen on dry ice and transferred to Shanghai Personal Biotechnology Co., Ltd., for WGS. Genomic DNA was extracted, purified, and fragmented to construct libraries with different insert sizes. A hybrid sequencing strategy utilizing both Illumina MiSeq (second-generation sequencing) and PacBio RS II (third-generation single-molecule real-time sequencing) platforms was implemented for comprehensive genome coverage.

### 2.4 Data quality control and assembly

Raw reads generated from both Illumina and PacBio sequencing platforms underwent quality assessment using FastQC (v0.20.0). Adapter sequences were trimmed, and low-quality reads (Phred quality score < 20) and short fragments (<50 bp) were filtered to retain high-quality clean reads. PacBio long reads were assembled into contigs using Unicycler v0.4.8 and Flye v2.7.1, followed by iterative polishing with Illumina short reads via Pilon v1.18 to generate a complete, high-accuracy genome sequence.

### 2.5 Gene annotation

Protein-coding sequences were annotated by homology searches against the Non-Redundant Protein Sequence (NR)^1^, Kyoto Encyclopedia of Genes and Genomes (KEGG)^2^, Gene Ontology (GO)^3^, Clusters of Orthologous Groups (COG)^4^, Carbohydrate-Active Enzymes (CAZy)^5^, Transporter Classification Database (TCDB)^6^, Virulence Factors of Pathogenic Bacteria (VFDB)^7^, and PhiSpy^8^ databases using Diamond (BLASTP mode), BLASTP, and HMMER (Hmmscan) algorithms.

### 2.6 Hemolytic activity assay

*Lacticaseibacillus rhamnosus* SAL2 and *Staphylococcus aureus* (positive control) were streaked onto blood agar plates and incubated at 37°C for 48 h and 24 h, respectively. Hemolytic activity was assessed by examining the plates for zones indicative of β-hemolysis (complete erythrocyte lysis), α-hemolysis (partial lysis with greenish discoloration), or γ-hemolysis (no lysis) (Zhang et al., 2021a).

### 2.7 Antibiotic susceptibility testing

The Kirby-Bauer disk diffusion method was performed, according to the guidelines and interpretive criteria established by the Clinical and Laboratory Standards Institute (CLSI M100-Ed35, 2025). Bacterial suspensions (adjusted to 0.5 McFarland standard) were uniformly spread on MRS agar plates using sterile swabs. Antibiotic disks were aseptically placed on the agar surface, and plates were incubated at 37°C for 48 h. Inhibition zone diameters were measured to determine susceptibility profiles.

### 2.8 High-salt tolerance assay

Seed cultures of *L. rhamnosus* SAL2 and *L. rhamnosus* GG were inoculated (10% v/v) into MRS broth containing 1%–8% (w/v) NaCl and incubated at 37°C with shaking (150 rpm) for 24 h. Survival rates were quantified via drop plate methods for enumeration. Survival rate was calculated as: Survival rate = Nt/N0 × 100%, where Nt = post-treatment CFU (log CFU/mL); N0 = initial CFU (log CFU/mL). Briefly, 200 μL of culture was serially diluted in sterile saline, and 20 μL aliquots were spotted onto MRS agar plates in triplicate. After 20 h of incubation at 37°C, viable counts (log CFU/mL) were calculated.

### 2.9 Bovine bile salt tolerance assay

Strains were exposed to MRS broth supplemented with 0.1%–0.4% (w/v) bovine bile salt for 2–4 h. Survival rates were determined as described for the high-salt tolerance assay (Lin et al., 2020).

### 2.10 Simulated gastric/intestinal fluid resistance

Simulated gastric fluid (pH 1.5, containing 0.3% pepsin) and intestinal fluid (pH 6.8, containing 0.1% pancreatin) were prepared according to the Chinese Pharmacopeia (2015 edition). Strains were incubated in simulated gastric fluid for 1 h, 2 h, 4 h and simulated intestinal fluid for 2 h, 4 h, 6 h, respectively. Survival rates were quantified using the drop plate method (Epecka et al., 2023).

### 2.11 Sample preparation

Individual colonies of *L. rhamnosus* SAL2 and *L. rhamnosus* GG were aseptically picked and inoculated into MRS broth medium. The cultures were incubated aerobically at 37°C with agitation (150 rpm) for 16 h, 24 h, 48 h, and 72 h. Post-incubation, bacterial cells were harvested by centrifugation (4,000 × *g*, 15 min, 4°C), and the resultant supernatant was collected as the fermented supernatant. The cell pellets were washed twice with sterile physiological saline (0.85% NaCl) and resuspended in fresh sterile saline to achieve a standardized bacterial suspension.

### 2.12 Adhesion assays

#### 2.12.1 Autoaggregation

The bacterial suspensions (OD_600_ = 0.6 ± 0.05) were vortexed for 30 s and statically incubated at 37°C for 24 h. Autoaggregation capacity was calculated as: Autoaggregation = (1–A24/A0) × 100%, where A0 = initial absorbance; A24 = absorbance after 24 h (Ma et al., 2023).

#### 2.12.2 Hydrophobicity

The bacterial suspensions (3 mL) were mixed with xylene (3 mL, 1:1 v/v), vortexed for 2 min, and phase-separated at room temperature for 30 min. We measured hydrophobicity via aqueous phase absorbance at 600 nm: Hydrophobicity = (1–A30/A0) × 100% (Tian et al., 2025).

### 2.13 Antioxidant activity assays

#### 2.13.1 H_2_O_2_ tolerance

Cells were harvested (8,000 × g, 5 min), washed twice with saline, and resuspended in MRS broth containing 1–10 mM H_2_O_2_. After 1 h of incubation at 37°C, survival rates were quantified using the drop plate method.

#### 2.13.2 DPPH radical scavenging activity

A sample (3 mL) was mixed with 0.2 mM DPPH ethanol solution (3 mL), incubated in darkness for 30 min, and centrifuged (4,000 × *g*, 10 min). Absorbance at 517 nm was measured to calculate scavenging capacity: DPPH scavenging = [1–(A_*sample*_–A_*blank*_)/A_*control*_] × 100% (Shi et al., 2019).

#### 2.13.3 Hydroxyl radical scavenging activity

Reaction mixtures containing 1 mL o-phenanthroline (2.5 mM), 1 mL PBS (pH 7.4), 1 mL FeSO_4_ (2.5 mM), 1 mL sample, and 1 mL H_2_O_2_ (20 mM) were incubated at 37°C for 1.5 h. Absorbance at 536 nm was measured after centrifugation (4,000 × *g*, 10 min): Hydroxyl scavenging = (A_*sample*_–A_*blank*_)/(A_*control*_–A_*blank*_) × 100% (Hu et al., 2019).

#### 2.13.4 Superoxide anion radical scavenging activity

Samples (0.1 mL) were mixed with 4.5 mL Tris-HCl (50 mM, pH 8.2) and 0.4 mL pyrogallol (25 mM, pre-warmed to 25°C). Reactions were quenched with 0.1 mL HCl (8 M) after 4 min of incubation at 25°C. Absorbance at 325 nm was measured post-centrifugation (4,000 × *g*, 10 min): Superoxide scavenging = {[A_*blank*_–(A_*sample*_–A_*control*_)]/Ablank} × 100% (Yu et al., 2024).

### 2.14 Statistical analysis

All experimental data were derived from a minimum of three independent biological replicates and are presented as mean ± standard deviation (SD). Prior to inferential analysis, data were assessed for normality and homogeneity of variance using Shapiro-Wilk and Levene’s tests, respectively. Statistical comparisons between the two strains across different experimental conditions were performed using two-way analysis of variance (ANOVA), with strain and treatment condition as the two independent factors. Where a significant main effect or interaction was identified (*P* < 0.05), *post hoc* pairwise comparisons were conducted using Tukey’s honestly significant difference (HSD) test.

For direct comparisons between the two strains at a single condition or time point, unpaired two-tailed Student’s *t*-tests were employed. The threshold for statistical significance was set at α = 0.05. Significance levels are denoted as follows: ns (not significant, *P* > 0.05), * (*P* < 0.05), ** (*P* < 0.01), *** (*P* < 0.001), and **** (*P* < 0.0001). All statistical analyses and graphical generation were performed using GraphPad Prism 9.0.0 (San Diego, California, USA).

## 3 Results

### 3.1 Whole genome sequencing analysis and function mining of *L. rhamnosus* SAL2

The genome of *L. rhamnosus* SAL2 comprises a single circular chromosome (2,989,570 bp; GC 46.76%) with no plasmids. A total of 2,767 open reading frames (ORFs) were predicted, collectively covering 2,529,300 bp.

**FIGURE 1:**
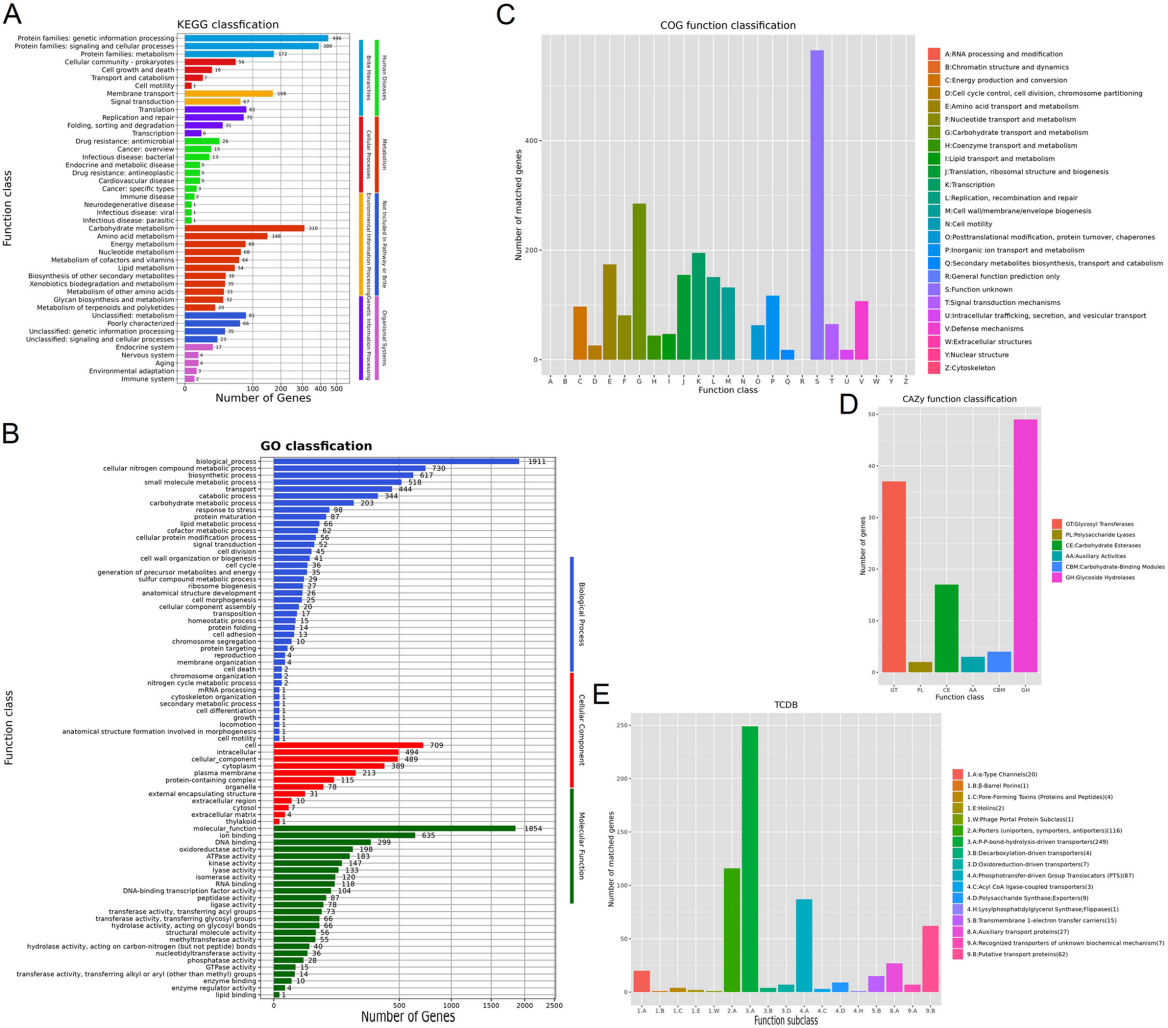
Functional classification of the *L. rhamnosus* SAL2 genome. **(A)** KEGG database annotation functional classification. **(B)** GO database annotation functional classification. **(C)** COG database annotation functional classification. **(D)** CAZy database annotation functional classification. **(E)** TCDB database annotation functional classification.

Kyoto Encyclopedia of Genes and Genomes pathway analysis classified 1,403 annotated genes (50.71% of total ORFs) into functional categories (Figure 1A), with carbohydrate metabolism representing the largest group (310 genes), followed by membrane transport (168 genes) and amino acid metabolism (148 genes). Gene Ontology (GO) analysis assigned 2,005 genes (72.46% of total ORFs) to biological processes, molecular functions, and cellular components (Figure 1B). Among biological processes, small molecule metabolic processes (518 genes), biosynthetic processes (617 genes), and cellular nitrogen compound metabolism (730 genes) were predominant. Key molecular functions included oxidoreductase activity (198 genes), DNA binding (299 genes), and ion binding (635 genes). Clusters of Orthologous Groups (COG) classification categorized 2,340 genes (84.57% of total ORFs) into 18 functional groups (Figure 1C), dominated by carbohydrate transport and metabolism (285 genes), with transcription-related genes (195 genes) and amino acid transport and metabolism (174 genes) as subsequent major categories.

The Carbohydrate-Active Enzymes (CAZy) database annotated 112 genes encoding carbohydrate-active enzymes (Figure 1D). Glycoside hydrolases (GHs) were the most abundant (49 genes), followed by glycosyl transferases (GTs, 37 genes) and carbohydrate esterases (CEs, 17 genes). Transporters Classification Database (TCDB) analysis identified 615 genes (22.23% of total ORFs) associated with transport systems (Figure 1E). P-P-bond-hydrolysis-driven transporters (249 genes) were predominant, followed by uniporters (116 genes), symporters (87 genes), and phosphotransfer-driven group translocators. Only one virulence-associated gene (gndA), linked to immune modulation and anti-phagocytic activity, was identified in the Virulence Factors Database (VFDB). This gene enables bacterial evasion of host immune defenses. PhiSpy software predicted eight prophage regions within the *L. rhamnosus* SAL2 genome, suggesting potential resistance to phage reinfection through lysogenic immunity (Tang et al., 2021).

Functional annotation highlighted a robust repertoire of antioxidant genes, including *msrA/B* (methionine sulfoxide reductases), *tpx* (thiol peroxidase), *trxA/B* (thioredoxins), and *btuE* (glutathione peroxidase). These genes encode enzymes critical for free radical scavenging, lipid peroxidation inhibition, and oxidative damage repair.

### 3.2 Safety evaluation of *L. rhamnosus* SAL2

**FIGURE 2:**
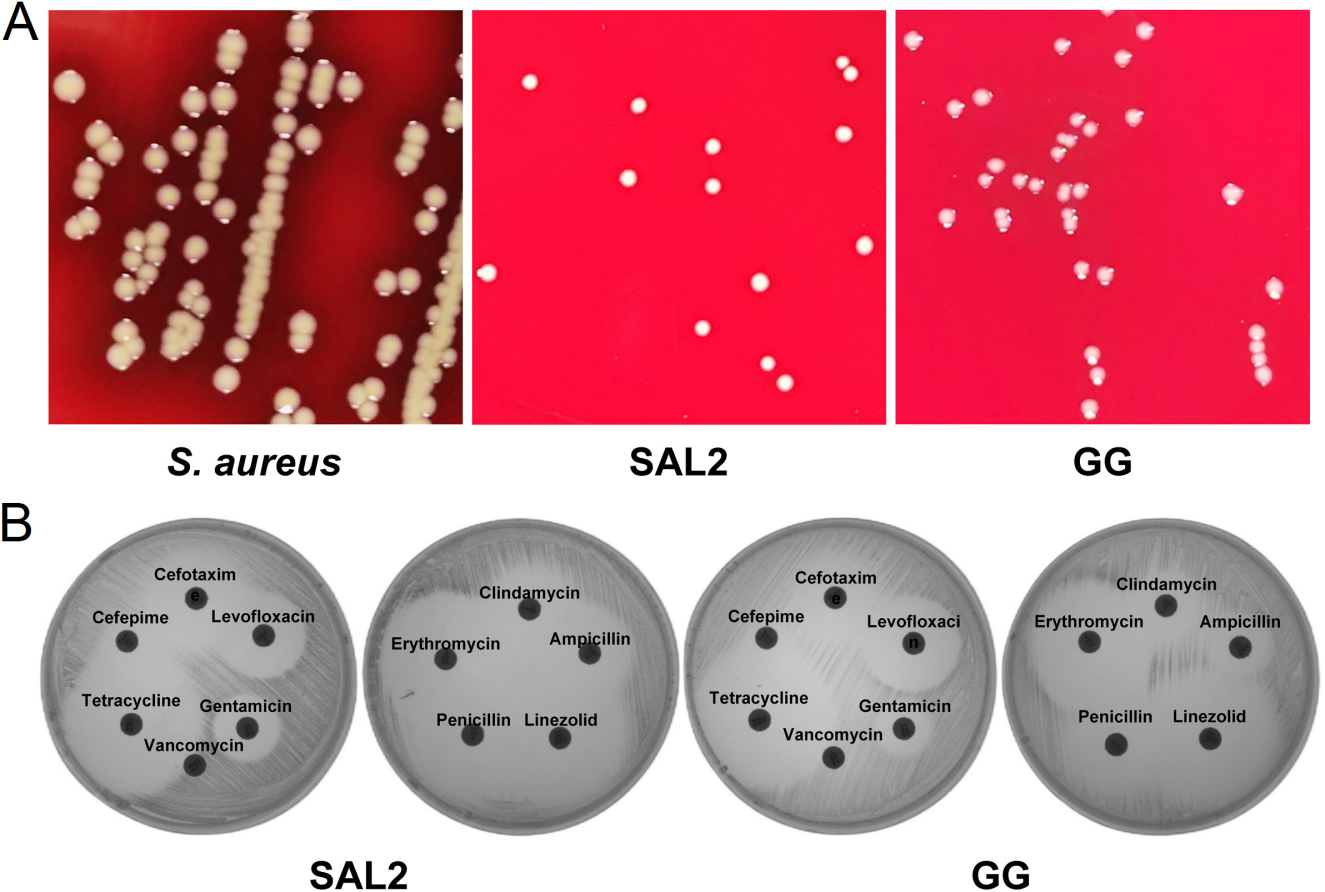
Safety evaluation of *L. rhamnosus* SAL2. **(A)** Hemolysis test results. **(B)** Antimicrobial susceptibility testing results.

*Staphylococcus aureus* showed β-hemolysis (clear zones), while neither *L. rhamnosus* SAL2 nor *L. rhamnosus* GG showed hemolytic activity on blood agar plates (Figure 2A). *L. rhamnosus* SAL2 demonstrated susceptibility to tetracycline, erythromycin, penicillin, ampicillin, gentamicin, clindamycin, cefotaxime, levofloxacin, and linezolid, intermediate susceptibility to cefepime, and resistance to vancomycin. In contrast, *L. rhamnosus* GG was resistant to penicillin, ampicillin, vancomycin, and cefepime, while showing intermediate susceptibility to cefotaxime (Figure 2B).

### 3.3 Probiotic characterization of *L. rhamnosus* SAL2

**FIGURE 3:**
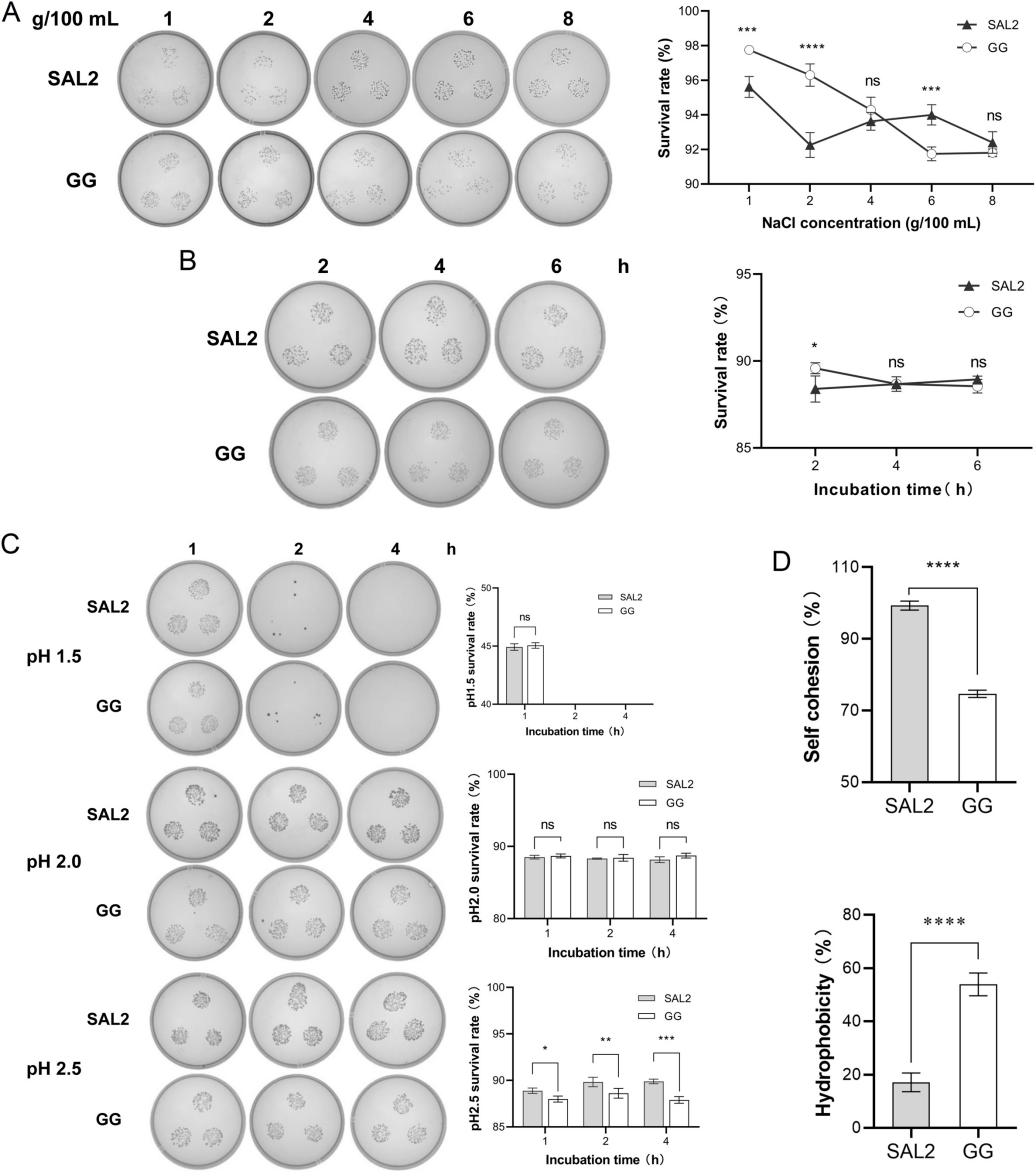
Probiotic characterization of *L. rhamnosus* SAL2. **(A)** Comparative analysis of high-salt tolerance between *L. rhamnosus* SAL2 and *L. rhamnosus* GG. Colony growth under varying NaCl concentrations both *L. rhamnosus* SAL2 and *L. rhamnosus* GG were serially diluted to 10^–5^ and cultivated on agar plates at different NaCl concentrations. Colony formation was assessed to evaluate salt tolerance. **(B)** Comparison of simulated intestinal fluid tolerance between *L. rhamnosus* SAL2 and *L. rhamnosus* GG. Colony growth in simulated intestinal fluid. *L. rhamnosus* SAL2 and *L. rhamnosus* GG were diluted to 10^–4^. **(C)** Comparison of simulated gastric fluid tolerance between *L. rhamnosus* SAL2 and *L. rhamnosus* GG. Colony growth profiles in simulated gastric fluid at different pH levels. pH 1.5: Undiluted cultures of *L. rhamnosus* SAL2 and *L. rhamnosus* GG. pH 2.0 and 2.5: Both strains were diluted to 10^–4^ prior to inoculation into simulated gastric fluid at pH 2.0 and pH 2.5. **(D)** Comparison of autoaggregation and cell-surface hydrophobicity between *L. rhamnosus* SAL2 and *L. rhamnosus* GG. (PS: *p < 0.05, **p < 0.01, ***p < 0.001, ****p < 0.0001, data were analyzed by two-way ANOVA).

*Lacticaseibacillus rhamnosus* GG showed higher survival rates than *L. rhamnosus* SAL2 at 1%–4% NaCl. However, under high-salt stress (6%–8% NaCl), *L. rhamnosus* SAL2 demonstrated significantly superior viability (*P* < 0.0001), with both strains maintaining viability >90% (Figure 3A). Growth of both *L. rhamnosus* SAL2 and *L. rhamnosus* GG was inhibited in media containing 0.1%–0.4% bovine bile salt, indicating limited bile tolerance. *L. rhamnosus* SAL2 showed increasing viability over 6 h in simulated intestinal fluid, whereas *L. rhamnosus* GG viability declined after 4 h. Both strains survived at >87% efficiency (Figure 3B). Both strains retained viability (>80%) in simulated gastric fluid at pH 2.0–2.5 but failed to survive beyond 2 h at pH 1.5 (Figure 3C). Autoaggregation capacity was significantly higher in *L. rhamnosus* SAL2 (99.27% ± 1.26%) compared to *L. rhamnosus* GG (74.65% ± 1.02%). Conversely, *L. rhamnosus* GG showed greater hydrophobicity (53.88% ± 4.31%) than *L. rhamnosus* SAL2 (17.09% ± 3.53%) (*P* < 0.0001) (Figure 3D).

### 3.4 Antioxidant activity of *L. rhamnosus* SAL2

**FIGURE 4:**
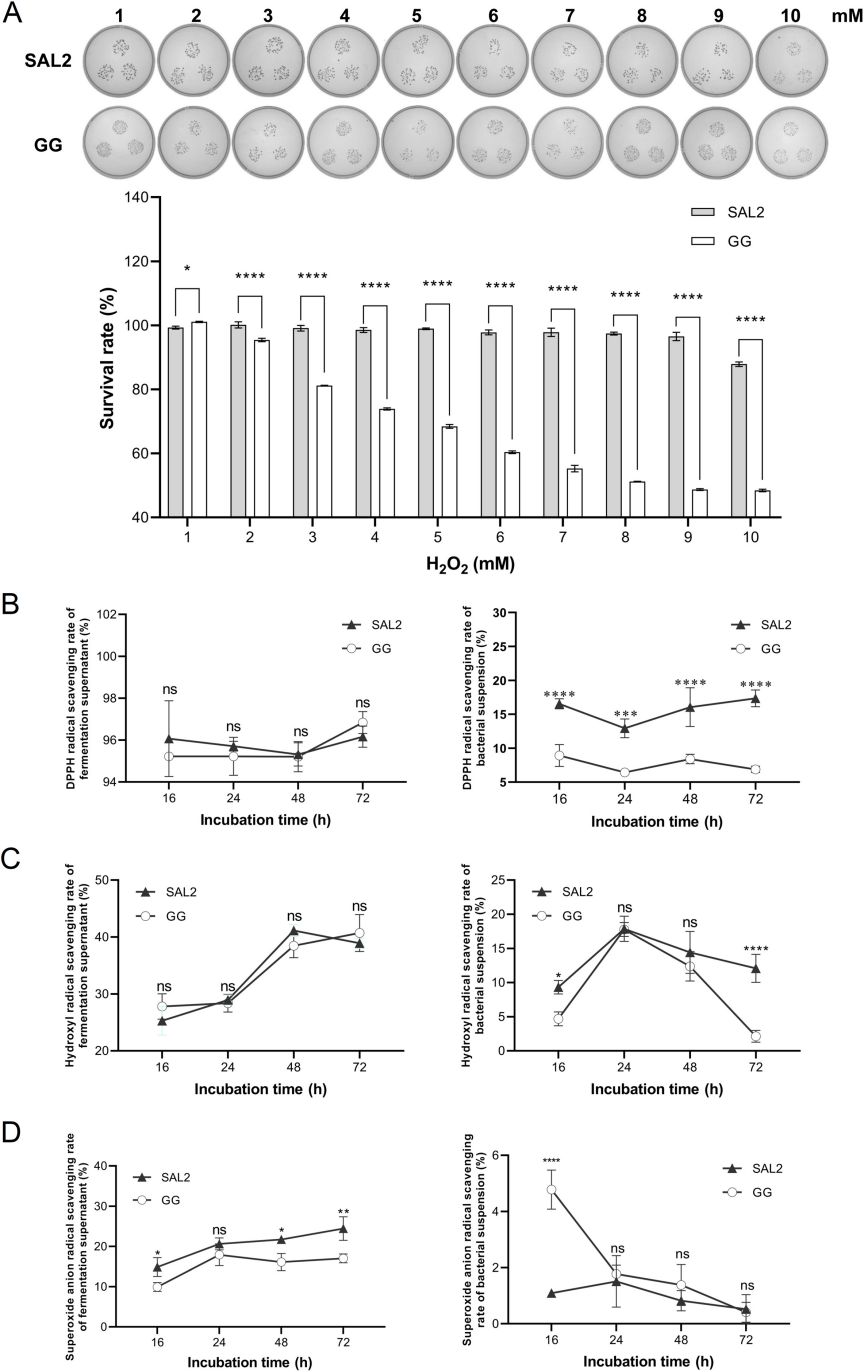
Antioxidant activity of *L. rhamnosus* SAL2. **(A)** Comparative analysis of H_2_O_2_ tolerance between *L. rhamnosus* SAL2 and *L. rhamnosus* GG. Colony growth under varying H_2_O_2_ concentrations. *L. rhamnosus* SAL2 was diluted to 10^–4^ at 1–9 mM H_2_O_2_ and to 10^–3^ at 10 mM H_2_O_2_. *L. rhamnosus* GG was diluted to 10^–4^ (1–2 mM H_2_O_2_), 10^–3^ (3 mM H_2_O_2_), 10^–2^ (4–7 mM H_2_O_2_), and remained undiluted (8–10 mM H_2_O_2_). **(B)** Comparative study of DPPH radical scavenging capacity between *L. rhamnosus* SAL2 and *L. rhamnosus* GG. **(C)** Comparison of hydroxyl radical scavenging capacity between *L. rhamnosus* SAL2 and *L. rhamnosus* GG. **(D)** Comparative analysis of superoxide anion radical scavenging abilities between *L. rhamnosus* SAL2 and *L. rhamnosus* GG. (PS: *p < 0.05, **p < 0.01, ***p < 0.001, ****p < 0.0001, data were analyzed by two-way ANOVA).

*Lacticaseibacillus rhamnosus* SAL2 maintained >95% survival at 1–9 mM H_2_O_2_, with a minimal decline to 87.90% ± 0.71% at 10 mM. In contrast, *L. rhamnosus* GG showed a sharp reduction in survival from 100% (1 mM) to 48.44% ± 0.41% (10 mM). Notably, *L. rhamnosus* SAL2 showed significantly higher tolerance than *L. rhamnosus* GG at concentrations ≥2 mM H_2_O_2_ (*P* < 0.0001) (Figure 4A).

Both strains showed stable DPPH radical scavenging rates across cultivation periods (Figure 4B). Cell-free fermentation supernatants showed significantly higher activity (>94%) compared to cell suspensions, with *L. rhamnosus* SAL2 cell suspensions outperforming *L. rhamnosus* GG (*P* < 0.0001). These results indicate that the majority of DPPH radical-scavenging compounds were localized in the cell-free fermentation supernatants. Cell-free fermentation supernatants of both strains showed superior hydroxyl radical scavenging activity compared to cell suspensions (Figure 4C). Peak activity was observed in *L. rhamnosus* SAL2 cell-free fermentation supernatants at 48 h (41.13% ± 1.05%) and *L. rhamnosus* GG cell-free fermentation supernatants at 72 h (40.71% ± 3.25%). *L. rhamnosus* SAL2 cell suspensions showed marginally higher activity than *L. rhamnosus* GG, though differences were not statistically significant (*P* > 0.05). *L. rhamnosus* SAL2 cell-free fermentation supernatants showed progressive increases in superoxide anion scavenging activity over time, whereas *L. rhamnosus* GG cell-free fermentation supernatants showed fluctuating trends (Figure 4D). *L. rhamnosus* SAL2 cell-free fermentation supernatants consistently outperformed *L. rhamnosus* GG at all time points, with cell suspensions exhibiting negligible activity. This further confirms that radical-scavenging metabolites are predominantly secreted into the extracellular milieu.

## 4 Discussion

The *Lacticaseibacillus rhamnosus* SAL2 genome showed the highest abundance of genes associated with carbohydrate transport and metabolism in both COG and KEGG databases. Additionally, it harbored numerous genes related to translation, transcription, amino acid transport, and metabolism, reflecting its robust mechanisms for carbohydrate metabolism, protein synthesis, and cellular growth. These attributes are critical for its survival and competitiveness within the complex intestinal environment of cancer patients. TCDB and KEGG annotations further indicated that *L. rhamnosus* SAL2 predominantly employs active transport systems to efficiently uptake nutrients and expel metabolic byproducts, ensuring sustained proliferation.

The strain’s rich repertoire of carbohydrate-active enzymes (CAZymes), including glycoside hydrolases (GHs) and glycosyl transferases (GTs), enables it to degrade diverse carbohydrates and synthesize complex polysaccharides. GHs, pivotal for hydrolyzing glycosidic bonds in substrates like lactose and cellulose, provide essential energy and carbon sources (Wardman et al., 2022). GTs, crucial for polysaccharide biosynthesis, facilitate the transfer of glycosyl groups to proteins, lipids, or other glycans, contributing to cell wall integrity and extracellular matrix formation (Gilfix, 2019; Gustaw et al., 2021). Annotation revealed a high proportion of genes associated with core metabolic functions. KEGG analysis indicated a major role in carbohydrate metabolism. Consistently, CAZy database annotation identified numerous genes encoding carbohydrate-active enzymes, with glycoside hydrolases (GHs) being the most abundant category, reflecting the strain’s capacity for carbohydrate utilization. Oxidative stress, driven by excessive reactive oxygen species (Borodovich et al., 2022), damages cellular components and is implicated in pathologies such as cardiovascular diseases, malignancies, and diabetes (Liu, 2020). *L. rhamnosus* SAL2 possesses a high proportion of oxidoreductase-encoding genes (GO annotation), which are vital for free radical scavenging, redox homeostasis, and cellular signaling (Huang et al., 2022; Nishida-Tamehiro et al., 2022; Smith and Moran, 2024). Its antioxidant gene cluster, including pathways for glutathione, methionine, and thioredoxin metabolism, not only fortifies intracellular defenses but may also modulate host immune responses by influencing redox states.

The virulence-associated gene was only annotated in *L. rhamnosus* SAL2. G6PD, a key enzyme in the pentose phosphate pathway, which generates NADPH to counteract ROS-induced oxidative stress (Zhang et al., 2021b; Ahamed et al., 2023). While pathogenic bacteria use *gndA* for immune evasion, its retention in *L. rhamnosus* SAL2 likely enhances niche adaptation and oxidative stress resilience. Furthermore, eight intact prophage regions identified in the genome could modulate genetic plasticity, biofilm development, and adhesion efficacy (Borodovich et al., 2022), potentially facilitating gut colonization under oxidative stress conditions (Molina-Quiroz et al., 2020).

Hemolytic activity, a critical safety indicator for probiotics, was undetectable in both *L. rhamnosus* SAL2 and the reference strain GG. Intrinsic resistance to vancomycin and cephalosporins, common in lactobacilli, is chromosomally encoded and non-transferable, aligning with guidelines from the European Food Safety Authority (EFSA) and Clinical and Laboratory Standards Institute (CLSI) (Das et al., 2020; Rozman et al., 2020; Stogios and Savchenko, 2020; Ojha et al., 2022). The absence of plasmids in *L. rhamnosus* SAL2 further minimizes horizontal gene transfer risks. Such resistance traits may enhance survival in antibiotic-rich environments, supporting gut microbiota equilibrium. Future studies will delineate the genetic basis of these resistance profiles and their clinical implications.

Under hyperosmotic stress, bacterial cells undergo dehydration due to rapid osmotic shifts, leading to cytoplasmic instability and metabolic dysfunction. Both *L. rhamnosus* SAL2 and *L. rhamnosus* GG demonstrated robust high-salt tolerance, with *L. rhamnosus* SAL2 exhibiting superior survival rates at elevated NaCl concentrations (6%–8%). Lactobacilli counteract osmotic stress by synthesizing compatible solutes (e.g., betaines, amino acids) to balance intracellular osmolarity, thereby preserving enzymatic activity and metabolic stability (Tang and Lu, 2019). Glycolytic enzyme systems and heat shock response pathways further contribute to salt tolerance, with strain-specific genomic adaptations explaining observed differences (Gao et al., 2022). The gastric barrier, mediated by acidic pH (1.5–3.0) and pepsin activity, challenges bacterial survival. Both strains showed ≥85% viability in simulated gastric fluid (pH 2.0–2.5) but failed to survive beyond 2 h at pH 1.5. In the small intestine, bovine bile salt and pancreatic enzymes exert antimicrobial effects via membrane disruption and DNA damage. While *L. rhamnosus* SAL2 and *L. rhamnosus* GG showed limited bile tolerance, *L. rhamnosus* SAL2 showed progressive viability increases in simulated intestinal fluid, suggesting enhanced stress adaptation or repair mechanisms compared to *L. rhamnosus* GG (Wu et al., 2012; Urdaneta and Casadesús, 2017).

Bacterial adhesion involves nonspecific (e.g., hydrophobicity) and specific (e.g., surface proteins, exopolysaccharides) interactions. *L. rhamnosus* SAL2 showed near-complete autoaggregation (99.27% ± 1.26%), significantly surpassing *L. rhamnosus* GG (74.65% ± 1.02%), a trait often correlated with enhanced potential for mucosal adhesion and biofilm formation *in vitro* (Suvarna et al., 2018; Kocabay and Çetinkaya, 2020; Reuben et al., 2020). Conversely, *L. rhamnosus* GG’s higher hydrophobicity (53.88% ± 4.31% vs. 17.09% ± 3.53% for *L. rhamnosus* SAL2) implies greater affinity for hydrophobic substrates, potentially enhancing epithelial binding (Rozman et al., 2020).

*Lacticaseibacillus rhamnosus* SAL2 outperformed *L. rhamnosus* GG in H_2_O_2_ tolerance, likely due to upregulated antioxidant genes (e.g., thioredoxin, glutathione systems) (Liu et al., 2019; Zhang et al., 2020). Cell-free fermentation supernatants of both strains showed superior radical-scavenging activity (>94% for DPPH, 41.13% for hydroxyl radicals, and progressive superoxide anion neutralization in *L. rhamnosus* SAL2), attributed to secreted antioxidants like exopolysaccharides, phenolics, and metal-chelating agents (Feng and Wang, 2020; Borisov et al., 2021; Ali et al., 2023). Cell suspensions showed modest activity, underscoring the extracellular localization of key antioxidants.

In conclusion, *L. rhamnosus* SAL2 demonstrates exceptional environmental resilience, safety, and probiotic potential. Its high-salt and oxidative stress tolerance, coupled with better adhesion potential and antioxidant secretion, position it as a promising candidate for gut microbiota modulation. The strain’s exopolysaccharides, likely central to its antioxidant efficacy, warrant further structural and functional characterization to explore their therapeutic applications as postbiotic agents in inflammation, immunity, and metabolic disorders. Ongoing studies will elucidate the genetic and metabolic underpinnings of these traits, advancing their translation into clinical and functional food contexts.

In conclusion, the test strain *L. rhamnosus* SAL2 is a promising probiotic candidate characterized by high safety and robust probiotic properties. Its biological activity is comparable to that of the well-established strain *L. rhamnosus* GG, with superior antioxidant activity. Whole-genome sequencing analysis reveals that *L. rhamnosus* SAL2 exhibits excellent viability and safety, as well as strong capabilities in carbohydrate metabolism, environmental adaptation, antioxidant activity, and secretion of extracellular polysaccharides.

## Data Availability

The datasets presented in this study can be found in online repositories. The names of the repository/repositories and accession number(s) can be found below: https://ngdc.cncb.ac.cn, CRA027822.

## Footnotes

1
https://www.ncbi.nlm.nih.gov/refseq/

2
https://www.genome.jp/kegg/

3
http://geneontology.org/

4
https://www.ncbi.nlm.nih.gov/research/cog/

5
http://www.cazy.org/

6
https://www.tcdb.org/

7
http://www.mgc.ac.cn/VFs/

8
https://github.com/linsalrob/PhiSpy
